# BMCC1, which is an interacting partner of BCL2, attenuates AKT activity, accompanied by apoptosis

**DOI:** 10.1038/cddis.2014.568

**Published:** 2015-01-22

**Authors:** Y Tatsumi, R Takano, M S Islam, T Yokochi, M Itami, Y Nakamura, A Nakagawara

**Affiliations:** 1 Division of Biochemistry and Innovative Cancer Therapeutics, Chiba Cancer Center Research Institute, Chuoh-ku, Chiba 260-8717, Japan; 2 Children's Cancer Research Center, Chiba Cancer Center Research Institute, Chuoh-ku, Chiba 260-8717, Japan; 3 Division Pathology, Chiba Cancer Center, Chuoh-ku, Chiba 260-8717, Japan

## Abstract

*B*NIP2 and Cdc42GAP homology (*B*CH) *m*otif-*c*ontaining molecule at the *c*arboxyl-terminal region *1* (*BMCC1*) gene is highly expressed in patients with favorable neuroblastoma (NB). It encodes a 340-kDa protein with a conserved BCH scaffold domain that may regulate signaling networks and multiple cellular functions, including apoptosis. In this study, we determined the mechanism by which BMCC1 promotes apoptosis in human NB and non-NB cells, as BMCC1 is normally expressed in various organs, particularly in neuronal and epithelial tissues. We demonstrated in this report that BMCC1 was induced by DNA damage, one of the triggers of intrinsic apoptosis. Accordingly, we investigated whether BMCC1 expression impacts intracellular signals in the regulation of apoptosis via its C-terminal region containing BCH scaffold domain. BMCC1 decreased phosphorylation of survival signals on AKT and its upstream kinase PDK1. BMCC1 upregulation was correlated with the activation of forkhead box-O3a (FOXO3a) (a downstream inducer of apoptosis, which is suppressed by AKT) and induction of BCL2 inhibitor BIM, suggesting that BMCC1 negatively regulates phosphorylation pathway of AKT, resulted in apoptosis. In addition, we found that BNIP2 homology region of BMCC1 interacts with BCL2. Intrinsic apoptosis induced by DNA damage was enhanced by BMCC1 overexpression, and was diminished by knockdown of BMCC1. Taken together, we conclude that BMCC1 promotes apoptosis at multiple steps in AKT-mediated survival signal pathway. These steps include physical interaction with BCL2 and attenuation of AKT-dependent inhibition of FOXO3a functions, such as transcriptional induction of BIM and phosphorylation of ataxia telangiectasia-mutated (ATM) after DNA damage. We propose that downregulation of BMCC1 expression, which is frequently observed in unfavorable NB and epithelial-derived cancers, may facilitate tumor development by abrogating DNA damage repair and apoptosis.

Neuroblastoma (NB) is one of the most common childhood solid tumors, which arises from the sympathoadrenal lineage of neural crest cells.^1^ NBs are primarily classified into two groups, favorable (stages 1, 2 and 4S) and unfavorable (stages 3 and 4); the former tends to regress spontaneously. In contrast, patients with high-risk NB die because of the tumor despite multimodal therapy including chemotherapy.^2^ Accumulated evidence demonstrate that *MYCN* amplification,^3, 4^ *a*naplastic *l*ymphoma *k*inase (*ALK*) mutation or amplification^5, 6, 7, 8^ and downregulation of the gene encoding nerve growth factor receptor (*TrkA*)^9, 10, 11^ has critical roles in unfavorable NB. However, the molecular mechanism of spontaneous regression in NB remains unknown. To better understand this mechanism, we investigated genes differentially expressed among clinical samples obtained from patients with the favorable and unfavorable NB subsets.^12, 13^ We then identified several genes with unknown function, which were highly expressed in favorable NB, such as *UNC5D*^14^ and *S*rc *h*omology 2 domain containing *F* (*Shf*).^15^

*B*NIP2 and Cdc42GAP homology (*B*CH) *m*otif-*c*ontaining molecule at the *c*arboxyl-terminal region *1* (*BMCC1*) gene is specifically expressed at high levels in favorable NB samples, indicating that *BMCC1* expression may be a favorable prognostic factor for NB.^16^ Similarly, *BMCC1/KIAA0367* deregulation in primary NB was identified by an integrative meta-analysis.^17^ *BMCC1* encodes a 340-kDa protein that comprises several functional motifs as follows: kinesin-binding and coiled-coil domains, proline-rich region, P-loop and a BCH domain (Supplementary Figure S1a).^16, 18^ Proteins with the BCH domain act as scaffold that modulates morphogenesis, differentiation, motility and apoptosis by association with components of signaling networks.^18^

The BMCC1 C-terminal isoform BNIPXL binds to a small GTP-binding protein RhoA and Lbc, a RhoA-specific guanine nucleotide exchange factor (RhoGEF), through its BCH domain, leading to the inhibition of RhoA-dependent stress fiber formation and malignant transformation.^19^ BMCC1 promotes neural apoptosis induced by the depletion of nerve growth factor (NGF),^16^ possibly through the BNIP2 homology region containing BH3 homology domain. Amino-acid sequence of BNIP2 is highly homologous to the C terminus of BMCC1, and this region promotes apoptosis by binding to BCL2 through its BH3 homology domain (Supplementary Figures S1a and b).^20, 21^

Signaling through the phosphoinositide 3-kinase (PI3K)-AKT pathway has critical roles in cell survival and death. Receptor-activated PI3K phosphorylates PDK1 at S241 and AKT at T308. Also, activated PDK1 phosphorylates AKT at the amino-acid residue T308. AKT suppresses mitochondrial apoptosis through phosphorylation-dependent inactivation of proapoptotic factors BAD^22^ and FOXO3a (forkhead box-O3a),^23^ a forkhead box-O transcription factor that contributes to apoptosis induced by BIM.^24^ Moreover, hyperactivation of AKT pathway occurs frequently in cancers,^25^ and increased AKT phosphorylation correlates with poor prognosis in NB.^23, 26^ However, extremely little is known about the mechanism with which BMCC1 promotes apoptosis and modulates signaling pathway via the BNIP2 homology region.

In this study, we investigated the role of BMCC1 in apoptosis to understand how BMCC1 expression contributes to improved prognosis and drug sensitivity of cancer, particularly in NB. We report that BMCC1 negatively regulates prosurvival AKT signaling pathway through the BNIP2 homology region containing BCH domain. BMCC1 is a key facilitator of mitochondrial apoptosis through the survival signal pathway activated by attenuation of AKT and associates with BCL2 after induction by stimuli from apoptosis. Indeed, inhibition of BMCC1 expression resulted in a decrease in apoptosis induced by DNA damage. This result is consistent with consequence of the inhibition of FOXO3a functions, such as BIM induction and increased ataxia telangiectasia-mutated (ATM) phosphorylation, which depends on phosphorylation of AKT.

## Results

### DNA damage increases *BMCC1* expression

**Figure 1:**
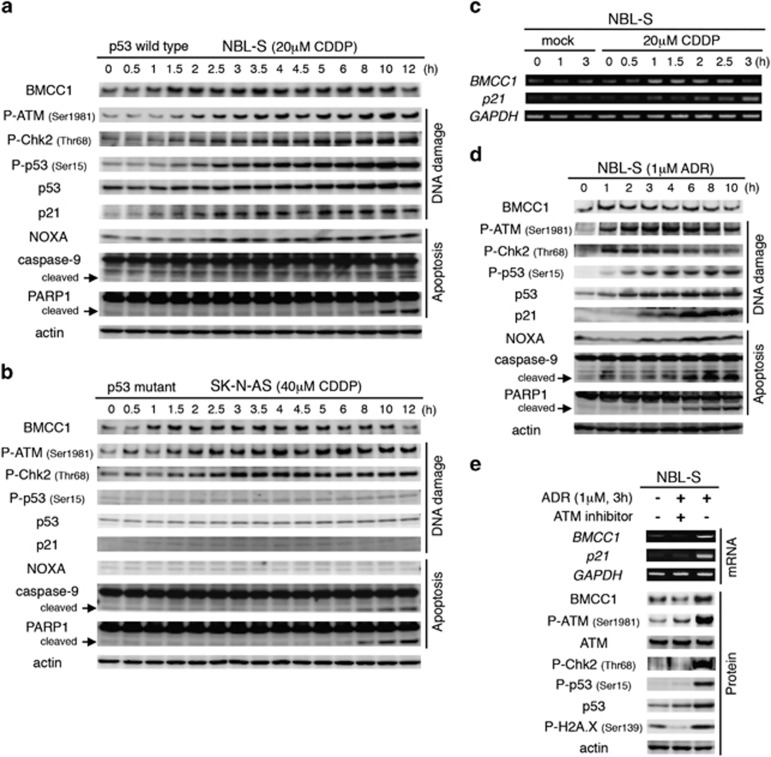
DNA damage-dependent induction of BMCC1 in NB cell lines. NBL-S (p53 wild-type, **a**) and SK-N-AS (p53 mutant, **b**) cells were treated with CDDP and were harvested at the times indicated. The cleaved forms of caspase-9 and PARP1 are indicated by arrows. (**c**) Mock- or CDDP-treated NBL-S cells were harvested at the times indicated, and mRNA expression was analyzed using semiquantitative RT-PCR. The glyceraldehyde 3-phosphate dehydrogenase (GAPDH) mRNA is used as a loading control. (**d**) Immunoblot analysis of BMCC1 expression in ADR-treated NBL-S cells. (**e**) ADR-dependent BMCC1 accumulation was abrogated when ATM activation was inhibited by an ATM inhibitor. Levels of mRNA (upper panel) and protein (lower panel) were analyzed by semiquantitative RT-PCR and immunoblotting, respectively

Sympathoadrenal cervical ganglions prepared from *BMCC1* transgenic mice undergo accelerated apoptosis induced by withdrawing NGF, and *BMCC1* is expressed in neuronal cells in which programmed cell death was induced by retinoic acid and NGF withdrawal.^16^ To understand the molecular role of BMCC1 in this process, we treated NB cells with DNA-damaging reagents. NBL-S cells (wild-type p53) were treated with cisplatin (CDDP) and subjected to immunoblotting (Figure 1a). Phosphorylation of ATM Ser1981 and its phosphorylation sites in Chk2 (Thr68) and p53 (Ser15) was determined. We detected p53 phosphorylation and induction of its target genes *p21* and *NOXA*. BMCC1 was detected 1 h after treatment before the onset of apoptosis, indicated by cleaved caspase-9 and poly (ADP-ribose) polymerase 1 (PARP1). Similarly, CDDP-mediated BMCC1 induction was observed in SK-N-AS cells (p53 mutant; Supplementary Figure S2c), indicating that *BMCC1* transcription occurred independently of p53 (Figure 1b). ATM and Chk2 phosphorylation was detected and not p53 phosphorylation or accumulation. Furthermore, induction of genes encoding p53-downstream signaling targets, such as p21 and NOXA, was undetectable in SK-N-AS cells. Semiquantitative RT-PCR analysis of CDDP-treated NBL-S cells indicated that BMCC1 was regulated at the transcriptional level (Figure 1c).

BMCC1 expression was significantly increased in NBL-S cells treated with adriamycin (ADR) and was accompanied by DNA damage response before the accumulation of cleaved caspase-9 and PARP1 (Figure 1d). To determine whether the induction of BMCC1 in NBL-S cells depended on the DNA damage response, we used an ATM inhibitor, which markedly decreased phosphorylation of ATM and its downstream substrates, such as Chk2, p53 and H2A.X (Figure 1e). The inhibition of ATM pathway was confirmed by transcription status of *p21*. Under this condition, inhibition of ATM yielded a decrease in transcription and translation of BMCC1, suggesting that phosphorylation of ATM is closely associated with the induction of BMCC1 after DNA damage.

### BMCC1 inhibits AKT phosphorylation

**Figure 2:**
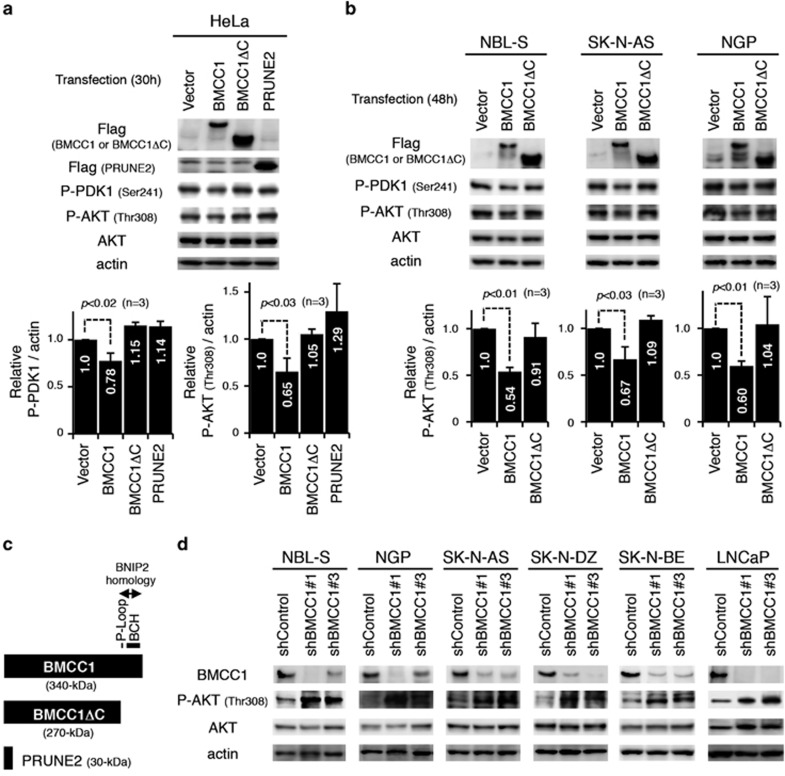
BMCC1 abrogated phosphorylation at T308 in AKT through the BNIP2 homology region. (**a**) Attenuated phosphorylation of PDK1-S241 and AKT-T308, mediated by BMCC1 overexpression, was detected in HeLa cells (upper panel). Mean values were calculated from triplicate experiments. Error bars indicate standard deviation. (**b**) BMCC1- and BMCC1ΔC-transfected NB cell lines were immunoblotted (upper panels). T308 phosphorylation of AKT was measured and mean values from triplicate experiments are represented (lower panels). (**c**) The structures of BMCC1, BMCC1ΔC and PRUNE2. BMCC1ΔC is the mutant lacking C-terminal P-loop and BNIP2 homology region. (**d**) BMCC1 knockdown using specific shRNAs induced phosphorylation of AKT in NB and LNCaP cells

Proteins containing BCH domain are thought to modulate signaling networks.^18^ Therefore, we examined whether BMCC1 negatively modulates AKT activity that promotes cell survival. We expressed BMCC1 in BMCC1-negative HeLa cells and LNCaP cells with AKT hyperactivation induced by *PTEN* (*phosphatase and tensin homolog*) mutation^27^ (Figures 2a and b and Supplementary Figures S3a and b). PTEN phosphatase is a component of the PI3K-AKT pathway. BMCC1-positive cells were used to determine the effects of inhibiting BMCC1 expression (Figure 2d).

BMCC1 overexpression in HeLa cells at the indicated time points showed reduced phosphorylation levels of PDK1-S241 and AKT-T308 compared with control cells (Supplementary Figure S3a), indicating that BMCC1 modulated the AKT pathway in epithelial cells. Decreased phosphorylation of PDK1-S241 and AKT-T308 in HeLa cells overexpressing BMCC1 was detected in the NB cells (Supplementary Figure S3b), indicating that BMCC1 inhibited PI3K-mediated phosphorylation of PDK1 and AKT. LNCaP cells overexpressing BMCC1 exhibited a similar phenotype, suggesting that inhibition of PDK1-S241 and AKT-T308 phosphorylation was independent of PTEN.

Next, we asked whether the BNIP2 homology region containing BCH domain in BMCC1 is responsible for the inhibition of AKT. We overexpressed full-length BMCC1 and BMCC1ΔC, which lacks the BNIP2 homology region and P-loop, as well as PRUNE2 (Figure 2c), in HeLa cells (Figure 2a). PDK1-S241 and AKT-T308 phosphorylation was significantly reduced in the cells overexpressing full-length BMCC1. In contrast, inhibition of PDK1 and AKT phosphorylation was not detected in cells overexpressing BMCC1ΔC and PRUNE2, suggesting that the BNIP2 homology region is required to inhibit the phosphorylation of AKT. Similar results were obtained using NB cells (Figure 2b). In contrast, BMCC1 depletion using shRNAs resulted in a significant increase in the phosphorylation of AKT-T308 (Figure 2d), indicating that lack of BMCC1 was required for the phosphorylation of AKT. Consistent with this notion, PDK1-S241 phosphorylation also increased slightly concomitant with the elevated phosphorylation of AKT-T308 in NB cells in which BMCC1 was depleted (Supplementary Figure S3c).

### BMCC1 promotes BIM expression by attenuation of the AKT-dependent phosphorylation of FOXO3a

Phosphorylation-dependent inhibition of FOXO3a mediated by activated AKT promotes cell survival, and deregulation of this pathway strongly associates with poor prognosis of NB.^23^ Because *BMCC1* expression is decreased in unfavorable NB,^16^ we investigated whether BMCC1 modulates proapoptotic FOXO3a activity when AKT activity was inhibited.

**Figure 3:**
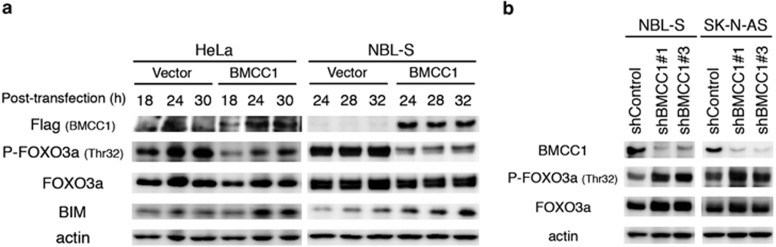
BMCC1 inhibited FOXO3a phosphorylation to induce proapoptotic BIM. (**a**) Reduced phosphorylation of FOXO3a-T32 and accelerated expression of BIM were detected in HeLa and NBL-S cells after BMCC1 overexpression. (**b**) BMCC1 knockdown using specific shRNAs increased FOXO3a-T32 phosphorylation in NBL-S and SK-N-AS cells

Phosphorylation level of FOXO3a-T32 decreased in HeLa and NBL-S cells, when BMCC1 was overexpressed (Figure 3a). Non-phosphorylated FOXO3a translocates to the nucleus to induce the transcription of target genes.^28^ FOXO3a accumulated in the nuclei of cells in which BMCC1 was overexpressed (Supplementary Figure S4a). BIM induction was subsequently detected (Figure 3a and Supplementary Figure S4b), which is consistent with reduced phosphorylation and increased FOXO3a nuclear localization. These data indicate that the BNIP2 homology region of BMCC1 enhanced the proapoptotic activity of FOXO3a after reduced phosphorylation of AKT. In contrast, an increase of FOXO3a phosphorylation was observed in NB cells treated with shRNAs that simultaneously depleted BMCC1 (Figure 3b) with elevated AKT phosphorylation (Figure 2d), indicating that BMCC1 modulates FOXO3a activity by abrogating the phosphorylation of AKT.

### Association of BMCC1 with BCL2 *in vivo*

Antiapoptotic activity of BCL2 was inhibited via BH3 domain.^29^ BH3 homology domain in BNIP2 was also reported to bind with antiapoptotic BCL2.^20, 21^ Because the C-terminal region of BMCC1, known as BNIP2 homology region, harbors a conserved BH3 homology domain (Supplementary Figure S1b),^16^ we confirmed whether BMCC1 associates with BCL2 *in vivo*.

**Figure 4:**
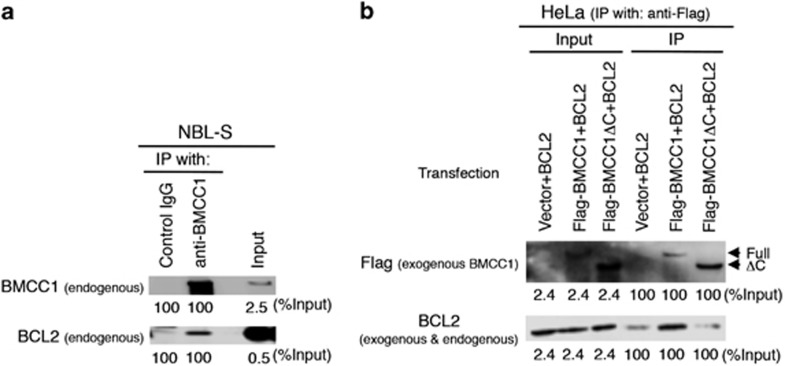
The C terminus of BMCC1 is responsible for binding to BCL2. (**a**) Immunoprecipitation analysis of the endogenous association between BMCC1 and BCL2 in NBL-S cells using an anti-BMCC1 antibody. The efficiency of immunoprecipitation is presented as the relative values (%Input). (**b**) Full-length BMCC1, and not BMCC1ΔC, was associated with BCL2 in HeLa cells. BCL2 and either Flag-tagged BMCC1 or BMCC1ΔC were overexpressed and immunoprecipitated using an anti-Flag antibody. Note that anti-BCL2 antibody recognizes both exogenous and endogenous BCL2 proteins

We proved the physical interaction between BMCC1 and BCL2 using endogenous proteins (Figure 4a) and overexpression system (Supplementary Figures S5a and b). Next, we sought to identify the binding region responsible for this interaction. Therefore, we examined whether the associations between BMCC1 and BCL2 was mediated by the BH3-containing BNIP2 homology region of BMCC1. Toward this end, we focused on the C terminus of BMCC1, which is homologous to BNIP2, to examine the interaction between BMCC1 and BCL2. Full-length BMCC1 or BMCC1ΔC and BCL2 were overexpressed in HeLa cells, and the cell lysates were subjected to immunoprecipitation (Figure 4b). The results revealed that full-length BMCC1, and not BMCC1ΔC, was associated with BCL2 *in vivo* through its C terminus containing BH3 domain.

It should be noted that endogenous BCL2 was co-precipitated with endogenous BMCC1 in a low efficiency (Figure 4a), possibly due to the interaction between BMCC1 and a small portion of BCL2 in the cytoplasm (Supplementary Figure S5c).

### BMCC1 activates intrinsic apoptosis

**Figure 5:**
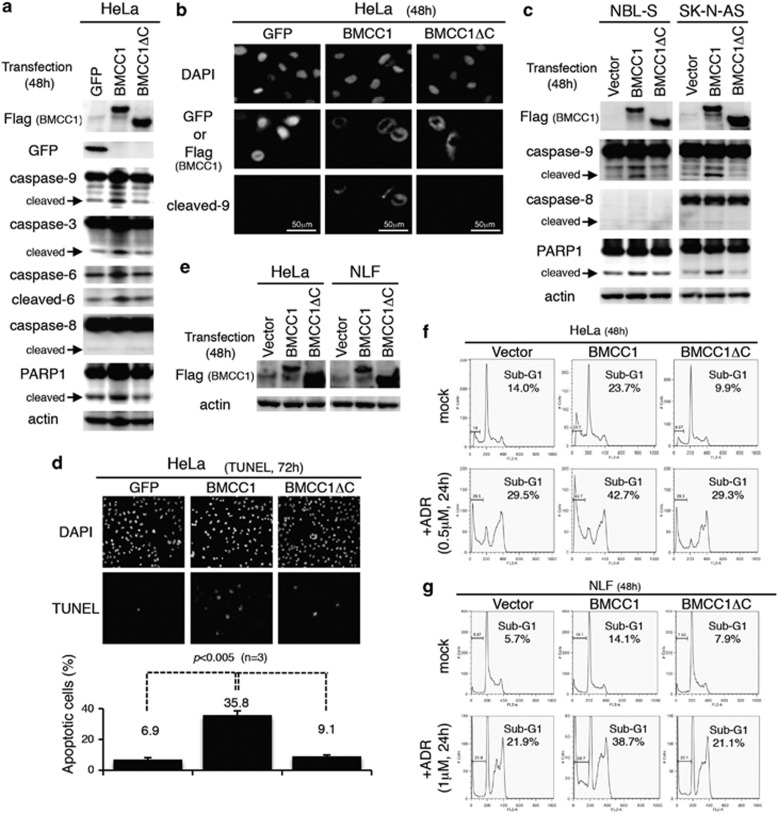
Activation of apoptotic pathway is mediated by the C-terminal BNIP2 homology region of BMCC1. (**a**) Immunoblot analysis of lysates prepared from HeLa cells 48 h after transfection. Cleaved caspase-9, caspase-3, caspase-8 and PARP1 are indicated by arrows. (**b**) Representative images of immunostaining using an antibody specific to cleaved caspase-9 (cleaved-9) are shown. Cleaved caspase-9 was detected only when full-length BMCC1 was overexpressed. (**c**) BMCC1 elevated the levels of cleaved caspase-9 and PARP1, whereas BMCC1ΔC did not. (**d**) Terminal deoxynucleotidyl transferase dUTP nick-end labeling (TUNEL) assay. Representative images are shown (upper panel), and a number of TUNEL-positive cells were counted (lower panel). The experiments were performed three times independently. Transfected HeLa (**f**) and NLF cells (**g**) were cultured for 48 h with or without ADR. Subsequent sub-G1 populations that include cells undergoing apoptosis were measured using FACS. Overexpression of BMCC1 and BMCC1ΔC was confirmed by immunoblot using anti-Flag antibody (**e**)

We demonstrated that BMCC1 inhibits AKT phosphorylation to induce BIM (Figures 2 and 3a and Supplementary Figure S4b) and interacts with BCL2 (Figure 4 and Supplementary Figure S5) through the BNIP2 homology region. Given the previous literatures reporting that BIM and BCL2 are pro- or antiapoptotic regulators,^30, 31^ we hypothesized that BMCC1 could promote intrinsic apoptosis using its BNIP2 homology region at the C terminus. Therefore, we used BMCC1 full-length and BMCC1ΔC constructs to prove this hypothesis (Figure 5).

First, we examined caspase activation mediated by BMCC1 or BMCC1ΔC overexpression. Immunoblotting revealed significant accumulation of cleaved caspase-9 in the cells expressing full-length BMCC1 after 48 h of transfection (Figure 5a). Furthermore, cleavage of caspase-3 and caspase-6, which are downstream substrates of activated caspase-9, was observed in the cells expressing full-length BMCC1. In contrast, BMCC1ΔC expression did not induce the cleavage of caspases as observed in the cells in which GFP was transfected. This is consistent with the result of immunostaining analysis, as cleaved caspase-9 was detected in the cells only when the full length of BMCC1 was overexpressed (Figure 5b). Accumulation of cleaved caspase-9 was also detected in the NB cells by the overexpression of full-length BMCC1 but not by BMCC1ΔC (Figure 5c). Therefore, we conclude that BMCC1, through the C-terminal region homologous to BNIP2, promoted activation of proteinase cascade initiated by caspase-9. It should be pointed out that caspase-8 was undetectable in NBL-S cells or, even when it was expressed, the cleavage of caspase-8 did not occur in HeLa or SK-N-AS cells overexpressing BMCC1 (Figures 5a and c). These data suggest that the C-terminal of BMCC1 is responsible for intrinsic apoptosis in a caspase-8-independent and mitochondria-dependent manner.

PARP1 cleavage, the consequence of caspases activation, was observed in the cells expressing full-length BMCC1, implying that apoptosis was induced in HeLa and NB cells (Figures 5a and c). Compared with GFP- or BMCC1ΔC-transfected cells, those expressing full-length BMCC1 showed significant increase in the number of TUNEL-positive cells (Figure 5d). FACS analyses also demonstrated apoptosis induction in the cells overexpressing full-length BMCC1 (Figures 5e–g). Accumulation of the sub-G1 population, a marker of apoptosis, was observed only in the cells overexpressing full-length BMCC1. These observations demonstrated that BMCC1 requires its BCH domain to induce apoptosis. Moreover, overexpression of full-length BMCC1, but not BMCC1ΔC, enhanced apoptosis induced by ADR. These results support our notion that BMCC1 activates the intrinsic apoptosis through the C-terminal domain.

### BMCC1 knockdown concurrently attenuates DNA damage response induced by DNA-damaging agents

**Figure 6:**
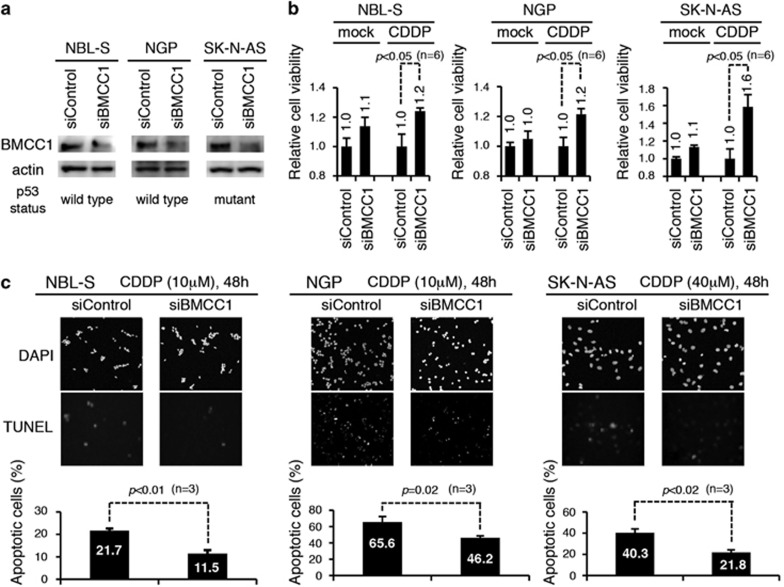
Attenuation of sensitivity to CDDP in NB cell lines transfected with BMCC1 siRNAs. (**a**) Immunoblot analysis to confirm BMCC1 knockdown mediated by specific siRNAs. (**b**) In the presence of CDDP, cell viability was significantly increased when BMCC1 expression was inhibited. Mean values of six experiments are shown. (**c**) NB cells transfected with BMCC1 siRNAs were treated with CDDP and were analyzed using TUNEL assay. Representative TUNEL images are shown (upper panel), and the mean values in the number of TUNEL-positive cells were plotted (lower panel)

As mentioned above, we showed that BMCC1 was induced after DNA damage (Figure 1) and BMCC1 overexpression increased the sensitivity of cells to DNA-damaging drugs (Figures 5e–g). Next, we sought to understand the role of BMCC1 followed by DNA damage. For this purpose, we used the strategy of siRNA knockdown. *BMCC1* mRNA expression was efficiently inhibited in the cells whose *p53* gene was either wild type (NGP and NBL-S) or mutated (SK-N-AS) (Figure 6a). Knockdown of BMCC1 efficiently increased the viability in NB cells after CDDP treatment, compared with the cells in which control siRNA was transfected (Figure 6b). Furthermore, the number of cells undergoing apoptosis induced by CDDP was significantly decreased by the inhibition of BMCC1 expression in NB cells (Figure 6c), suggesting that BMCC1 contributes to apoptosis induced by CDDP treatment regardless of the status of *p53*.

**Figure 7:**
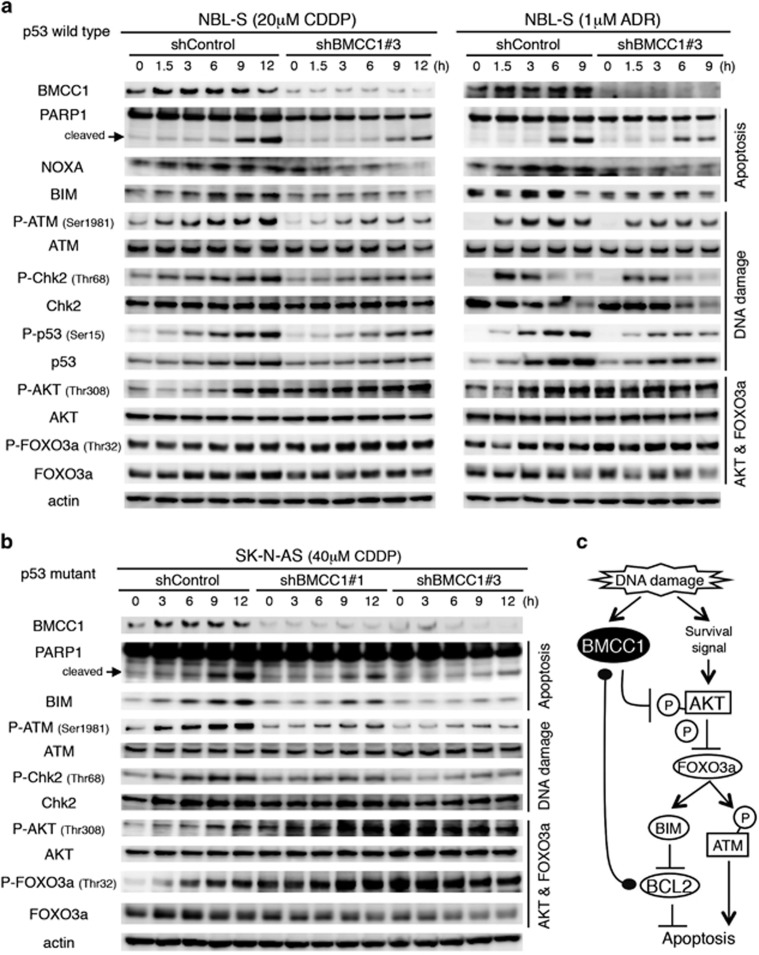
Downregulation of BMCC1 resulted in the attenuation of phosphorylation signals related to DNA damage response. (**a**) Phosphorylation signals induced by CDDP (left panel) or ADR (right panel) were decreased under the condition that BMCC1 was knocked down. BMCC1 knockdown resulted in increased phosphorylation at AKT-T308 and FOXO3a-T32 in NBL-S cells. (**b**) Phosphorylation signal induced by CDDP was decreased under the condition that BMCC1 was knocked down in SK-N-AS cells. (**c**) Proposed model of proapoptotic roles of BMCC1 at multisteps in AKT survival signal pathway

We further investigated apoptosis induced by either CDDP or ADR in the cells in which BMCC1 was knocked down (Figure 7). shRNA-mediated BMCC1 knockdown revealed a significant decrease in the expression levels of proapoptotic NOXA and BIM. In addition, PARP1 cleavage induced by CDDP or ADR was also decreased. These results suggest that apoptosis was inhibited by knockdown of BMCC1. Similar result was obtained in p53-mutated SK-N-AS cells treated by CDDP (Figure 7b).

BMCC1 knockdown in NB cells, in which apoptosis was inhibited, revealed significant reduction of phosphorylation at specific amino-acid residues in ATM and downstream targets, such as ATM-S1981, Chk2-T68 and p53-S15. This indicates that BMCC1 facilitates the signaling pathway of DNA repair, which was triggered by DNA-damaging reagents (Figure 7).

### BMCC1 downregulation in cancer tissues

**Figure 8:**
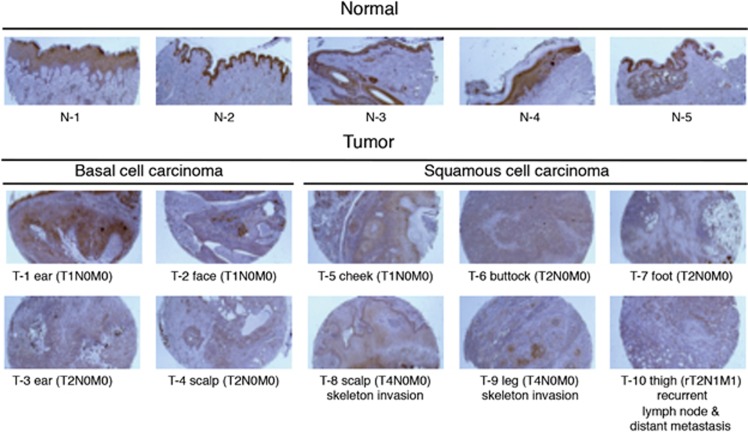
Reduced expression of BMCC1 in epithelial tissues in skin cancer. The expression levels of BMCC1 in cancer tissues were analyzed using an anti-BMCC1 antibody in immunohistochemistry (IHC). Tissue sections from epithelial-derived skin cancer (T=10 cases) and their corresponding normal tissues (N=5 cases) were shown. BMCC1 downregulation was significant in T2N0M0 tumors (T-3, T-4, T-6 and T-7), in T4N0M0 cases with skeletal invasion (T-8 and T-9) and in recurrent tumors (rT2N1M1) with lymph node and distant metastasis (T-10)

BMCC1 is frequently downregulated in unfavorable NB both at mRNA and protein levels.^16^ In this study, we detected ubiquitous BMCC1 expression in normal tissues (Supplementary Figures S2a and b). Therefore, we assessed whether BMCC1 expression detected in normal tissues, particularly in epithelium, was downregulated in tumors. We analyzed tissue sections from epithelial-derived skin, prostate, colon cancers and the corresponding normal tissues (Figure 8 and Supplementary Figure S6). Four basal cell carcinoma and six squamous cell carcinoma tissue sections were collected from various parts of the skin. Compared with the epithelia of normal skin (N-1 to N-5), BMCC1 expression was significantly reduced in tumors (T-1 to T-10) (Figure 8). We subsequently compared BMCC1 expression among five cases of relatively advanced prostate adenocarcinomas with that of epithelial cells of normal prostate tissue. Reduced BMCC1 staining was observed in all prostate tumor sections regardless of stage and Gleason score (Supplementary Figure S6a). Similar to skin and prostate cancers, decreased BMCC1 expression was detected in metastatic colon cancers regardless of the tumor type and origin (Supplementary Figure S6b). These data suggest that the expression level of BMCC1 was lower in epithelial-derived skin, prostate and colon cancers, including advanced cases resembling aggressive NB in which the expression level of BMCC1 was reduced.^16^

## Discussion

In this study, we demonstrated that BMCC1 induces apoptosis in human tumor cells, resulting in tumor suppression. BMCC1 binds to BCL2 through the BNIP2 homology region containing BH3 homology domain. The expression level of BMCC1 was increased by DNA damage, and BMCC1 inhibited phosphorylation of AKT, which is a crucial step in survival signaling pathway. BMCC1 overexpression contributed to mitochondrial apoptosis by caspase-9 activation. These results suggest that BMCC1 negatively regulates survival signal pathway through AKT. Given that BMCC1 is expressed at low levels in aggressive NB^16^ and other tumors such as skin, prostate and colon cancers, we propose that inhibition of the expression of BMCC1 may be associated with an increase of drug resistance, resulting in tumor progression. This assumption could be proved during the course of clinical study to ask whether BMCC1 expression correlates with response to practical chemotherapy or not. Hence, it is true that further investigation is required to better understand the role and significance of BMCC1 on the development of human cancer. Nonetheless, our finding in this manuscript clearly demonstrates that BMCC1 is one of key factors to gain insight into the molecular basis on cell survival and death.

BNIP family proteins possess a conserved BCH domain, which likely mediates diverse cellular functions.^18^ The BCH domain of BNIP2 binds to BCL2 and E1B, adenovirus BCL2 homolog and neutralizes their antiapoptotic functions.^20, 32^ Another BNIP family protein BNIP-S*α* functions in a similar manner.^21, 33^ In this study, we showed that BNIP2 homology region of BMCC1 was associated with BCL2 in the cytoplasm. BMCC1 induces BIM through the AKT-FOXO3a pathway. BIM binds to BCL2 and inhibits its survival activity in the mitochondria.^31, 34^ FOXO3a promotes the DNA damage response by an increase of ATM phosphorylation.^35, 36^ We indicated that BMCC1 depletion abrogated ATM phosphorylation and subsequent DNA damage response in association with AKT-dependent FOXO3a inhibition. ATM phosphorylation triggers the p53-dependent apoptotic pathway. This means alternative pathway of BMCC1 to promote apoptosis by the upregulation of p53 target genes. Expression of NOXA, mediated by p53, was inhibited by BMCC1 knockdown in the cells with wild-type p53. Therefore, we assume that BMCC1 inhibits cytoplasmic and mitochondrial BCL2 and other antiapoptotic BCL2 family proteins in DNA-damaged cells to ensure mitochondrial apoptosis (Figure 7c).

Among proapoptotic BNIP family members, only *BMCC1* expression is linked to NB prognosis.^16, 17^ In this study, we propose that BMCC1 promotes cellular signals of DNA damage repair and apoptosis at multiple steps in AKT survival signal pathway. Practically, deregulation of the AKT-FOXO3a pathway has a pivotal role in aggressive NB.^23^ Recent whole-genome sequence analysis of primary NBs revealed that a higher frequency of somatic mutation occurs only in stage 3 and stage 4 of aggressive NBs.^37^ Considering that the expression level of BMCC1 was very low in high-risk NB,^16^ such reduced expression of BMCC1 may mediate genomic instability by attenuation of DNA repair and apoptosis through the hyperactivation of AKT followed by the inhibition of FOXO3a.

TrkA-dependent apoptosis in neuronal cells is triggered by the depletion of NGF, resulted in spontaneous regression in NB.^10, 11, 38^ NGF depletion facilitates FOXO3a activity mediated by BIM through the negative regulation of PI3K-AKT signaling pathway.^24^ Therefore, BMCC1 may contribute to the spontaneous regression through AKT-FOXO3a regulation. The lack of data on transcriptional regulation of *BMCC1* prevents drawing conclusions regarding its role in apoptosis induced by NGF depletion. Although transcription factors such as p53,^14^ p63^39^ and E2F1^14, 40^ are involved in this process, transcription factors other than p53 may transcriptionally regulate BMCC1 in spontaneous regression, because, in this study, we indicated that BMCC1 was induced even in p53-defective cells after DNA damage.

We observed that BMCC1 inhibited AKT-T308 phosphorylation in LNCaP cells harboring mutated *PTEN*, indicating that the inhibition of AKT phosphorylation was mediated by negative regulation of the PI3K-AKT pathway independent of PTEN. The BCH domain in BMCC1 inhibits RhoA activity by binding to Lbc RhoGEF,^19^ and RhoA activates 1-phosphatidylinositol-4-phosphate 5-kinase (PIP5K) that is involved in the production of phosphatidylinositol (PtdIns)(3,4,5)*P*_3_ and its substrate PtdIns(4,5)*P*_2_ through Rho-associated, coiled-coil containing protein kinase (ROCK) activation,^41, 42, 43^ eventually promoting AKT phosphorylation. Therefore, BMCC1-RhoA-ROCK-PIP5K-AKT pathway might be a considerable way to explain the AKT inhibition through BCH domain of BMCC1. Another possible explanation for this regulation is that BMCC1 may regulate postendocytic trafficking by associating with adapter-related protein complex 2 (AP-2), a member of the endosomal protein complex, which contributes to the clathrin-dependent endocytotic internalization of receptors.^44^ Although precise BMCC1 function in endocytotic regulation was elusive, molecular mechanisms of endocytotic regulation or recycling of various receptors and subsequent modulation of retrograde signal transductions, including activation of the PI3K-AKT pathway, has been studied extensively.^45^ Notably, AKT is hyperactivated by downstream of deregulated TrkB^46^ and ALK^8, 47^ signaling pathway in aggressive NB. Therefore, future study will uncover the mechanism of how BMCC1 inhibits AKT phosphorylation.

The present study demonstrates the role of full-length BMCC1 in apoptosis induction. The C-terminal isoform of BMCC1 (BNIPXL) contributes to the reorganization of the actin cytoskeleton and inhibits malignant transformation,^19^ and the brain-specific C-terminal variant of BMCC1 (BMCC1s)^48^ functions in determining cell morphology. These isoforms possess the BCH domain. These diverse findings imply that the multiple functions of a large molecule such as BMCC1 may vary depending on cell type. It is assumed that other functions remain to be discovered. When such an understanding sheds light on the role of BMCC1 in the signal-transduction pathway, it will explain how BMCC1 maintains homeostasis and why BMCC1 expression is downregulated in cancer cells. In this study, the molecular functions of BMCC1 in the promotion of apoptosis and DNA damage repair provide clues for defining the underlying molecular mechanism(s) that determine whether the course of NB will be favorable or unfavorable.

## Materials and Methods

### Plasmid DNAs

*BMCC1* and *PRUNE2* cDNAs were amplified using PCR from human cDNA libraries containing a C-terminal 3 × Flag-tag sequence^49^ and were ligated into pENTR vector (Life Technologies, Carlsbad, CA, USA) using *Sal*I and *Not*I sites. Using the Gateway system (Life Technologies), *BMCC1* and *PRUNE2* were inserted into pcDNA6.2 (Life Technologies), yielding pcDNA6.2-BMCC1 (340-kDa) and pcDNA6.2-PRUNE2 (30-kDa) expression vectors. BMCC1ΔC (270-kDa) expression vector pcDNA6.2-BMCC1ΔC was constructed using C-terminal sequence of *BMCC1*, which was excised by *Kpn*I from the pcDNA6.2-BMCC1. The BCL2 expression plasmid pCDNA3-Bcl-2 was prepared from pCAGGS-Bcl-2 that was described previously.^50^ The pEGFP-N1 plasmid was purchased from Clontech Laboratories (Palo Alto, CA, USA).

### Cell culture, transfection and lentivirus infection

NB cell lines, SK-N-AS, NBL-S, NGP, NB9, NLF, SK-N-BE and SK-N-DZ, were obtained from the Children's Hospital of Philadelphia cell line bank (Philadelphia, PA, USA). These cells were maintained in RPMI-1640 (Life Technologies) supplemented with 10% heat-inactivated fetal bovine serum (Life Technologies), 100 IU/ml penicillin (Life Technologies) and 100 *μ*g/ml streptomycin (Life Technologies) in a humidified atmosphere containing 5% CO_2_ at 37 °C. HeLa, LNCaP, PC3 and 293T cells were cultured as described previously.^15, 16, 51^ Apoptosis was induced using CDDP (Sigma-Aldrich, St. Louis, MO, USA) or ADR (Kyowa Hakko Kirin Co., Tokyo, Japan). For ATM inhibition, 13 *μ*M of ATM kinase inhibitor (Calbiochem, San Diego, CA, USA) was used. For transient overexpression, cells were seeded onto 6-well plates and transfected with 2 or 4 *μ*g of expression vector with FuGENE HD (Promega, Fitchburg, WI, USA) or with Lipofectamine 2000 (Life Technologies), respectively. To inhibit endogenous *BMCC1* mRNA expression, human cells were transfected with 20 nmol/l *BMCC1*-specific siRNAs using HiPerFect Transfection Reagent (Qiagen, Valencia, CA, USA) or were infected with lentiviral *BMCC1* shRNAs. A mixture of three *BMCC1*-specific siRNAs (Integrated DNA Technologies, Coralville, IA, USA) was used for transfection. Their sequences are as follows: siBMCC1-1-S, 5′-GGAGAAGGAUAUUGACUUGAAGCTC-3′ and siBMCC1-1-AS, 5′-CAGCUUCAAGUCAAUAUCCUUCUCCAU-3′ siBMCC1-2-S, 5′-GGAGUAUCAGGAAGCAAAUCAGGTA-3′ and siBMCC1-2-AS, 5′-UACCUGAUUUGCUUCCUGAUACUCCAA-3′ and siBMCC1-3-S, 5′-CCCAGUGAGAUAAACAAUGAAGCAG-3′ and siBMCC1-3-AS, 5′-CUGCUUCAUUGUUUAUCUCACUGGGUG-3′. DS Scrambled Neg (Integrated DNA Technologies) served as a negative control. For lentiviral shRNA expression, MISSION shRNA plasmid DNA clones specific to *BMCC1/KIAA0367*, shBMCC1 no. 1 (TRCN0000156045) and shBMCC1 no. 3 (TRCN0000154470) were purchased from Sigma-Aldrich.

### Semiquantitative RT-PCR

To determine *BMCC1* mRNA expression levels, total RNA was isolated using the RNeasy Mini Kit (Qiagen) and was reverse-transcribed using random primers and SuperScript II reverse transcriptase (Life Technologies). The cDNA was PCR amplified using r*Taq* DNA polymerase (Takara Bio, Shiga, Japan). PCR primer sequences were used as follows: (forward and reverse): BMCC1-F, 5′-CTCATCACCGAGCAACTGGCTCATC-3′ and BMCC1-R, 5′-CACTGCCTGCCACGGCTTCTGTTG-3′ p21-F, 5′-GCGATGGAACTTCGACTT-3′ and p21-R, 5′-CAGGTCCACATGGTCTTCCT-3′ and GAPDH-F, 5′-ACCACAGTCCATGCCATCAC-3′ and GAPDH-R, 5′-TCCACCACCCTGTTGCTGTA-3′. *GAPDH* expression served as an internal control.

### Antibodies

A polyclonal antibody raised against a synthetic peptide representing an internal region of human BMCC1 was prepared in immunised rabbits (MBL, Nagoya, Japan) (Supplementary Figure S1a). The rabbit polyclonal antibody anti-PRUNE2 recognizes the N-terminal region of human BMCC1 and PRUNE2 (Proteintech Group, Chicago, IL, USA). Antibodies anti-PARP-1 (sc-8007), anti-ATM (2C1, sc-23921), anti-Bcl-2 (100, sc-509), anti-p53 (DO-1, sc-126), anti-p21 (sc-817), anti-tubulin (H-300, sc-5546) and anti-actin (sc-8432) were purchased from Santa Cruz Biotechnology Inc. (Santa Cruz, CA, USA). Anti-Akt (no. 9272), anti-phospho-Akt (Thr308, no. 9275), anti-phospho-PDK1 (Ser241, no. 3061), anti-phospho-p53 (Ser15, no. 9284), anti-phospho-ATM (Ser1981, no. 4526), anti-phospho-Chk2 (Thr68, no. 2661), anti-caspase-9 (no. 9502), anti-caspase-6 (no. 9762), anti-BIM (no. 4582, immunoblotting), anti-BIM (no. 2933, immunostaining), anti-phospho-FOXO1/FOXO3a (Thr24/Thr32, no. 9464), anti-FOXO3a (no. 2497), anti-lamin A/C (no. 2032) and HRP-conjugated anti-rabbit (no. 7074) and anti-nmuse (no. 7076) secondary antibodies were purchased from Cell Signaling Technologies (Danvers, MA, USA). Antibodies purchased from Abcam (Cambridge, MA, USA) were: anti-Noxa (ab13645) antibody and an antibody raised against cleaved caspase-9 (ab52299). Anti-phospho-H2A.X (Ser139, no. 07-164), anti-caspase-8 (AM-46) and anti-caspase-3 (no. 235412) were purchased from EMD Millipore (Billerica, MA, USA). Anti-Tim23 was from Becton Dickinson (BD; Franklin Lakes, NJ, USA), anti-Flag (M2) was from Sigma-Aldrich and anti-GFP (M048-3) was from MBL.

### Immunoblotting

Whole-cell lysates, prepared with SDS sample buffer containing protease (Nakalai Tesque, Kyoto, Japan) and phosphatase inhibitor cocktails (Calbiochem), were separated by SDS-PAGE and were transferred to PVDF membranes (Immobilon-P; Millipore). The membranes were incubated at room temperature with primary antibodies for 2 h and then with HRP-conjugated secondary antibodies for 1 h. The membranes were treated with ECL reagent (GE Healthcare, Buckinghamshire, UK) and signals were detected using an LAS-4000 Image Analyzer (GE Healthcare). ImageQuant TL software (GE Healthcare) was used to quantitate the intensity of bands. The Ready-to-Use Mouse Mixed Tissue Western Blot (Panel 5; Zyagen Laboratories, San Diego, CA, USA) PVDF membrane was used to analyze BMCC1 expression in mouse tissues.

### Immunoprecipitation and immunostaining

NBL-S cells, transfected LNCaP or HeLa cells were lysed in modified CSK buffer containing 0.1% Triton X-100 and protease and phosphatase inhibitors.^49^ The lysates were incubated with antibodies for 3 h at 4 °C. Immune complexes were precipitated with rotation by Protein G Sepharose beads (GE Healthcare) for 1 h at 4 °C. Beads were washed with 50 mM Tris-HCl (pH 7.4), 150 mM NaCl, 0.1% Triton X-100 and 1 mM EDTA four times at 4 °C. Immunoprecipitated proteins were eluted from the beads using 100 mM glycine-HCl (pH 2.5), boiled in SDS sample buffer and immunoblotted as described above. For immunostaining, cells were seeded onto coverslips and processed as described previously.^15^ Secondary antibodies used were: goat anti-rabbit IgG conjugated to Alexa Fluor 488 (Life Technologies) or Alexa Fluor 546; goat anti-mouse IgG antibody conjugated with Alexa Fluor 488 or Alexa Fluor 546. The stained images were analyzed using a Leica confocal microscope (Leica, Wetzlar, Germany).

### Cell viability, apoptosis detection and fractionation

Cell viability was measured using the WST-8 assay (Dojindo, Kumamoto, Japan). To detect apoptosis, flow cytometry (FACSCalibur; BD) was used after propidium iodide staining with a Cycle Test Kit (BD), and the number of cells in the sub-G1 stage was calculated using FlowJO (Tomy Digital Biology, Tokyo, Japan). In addition, TUNEL assay was performed to detect apoptosis in the cells transfected with BMCC1 or GFP expression vectors, or with *BMCC1* siRNAs. Cells were seeded onto coverslips and stained using the *In situ* Cell Death Detection Kit, TMR red (Roche). To isolate mitochondria, cells were lysed in a fractionation buffer (20 mM HEPES-HCl (pH 7.5), 10 mM KCl, 15 mM MgCl_2_, 1 mM EDTA, 1 mM EGTA, 250 mM sucrose), homogenized and centrifuged for 10 min at 800 × *g*. The supernatant was centrifuged for 15 min at 10 000 × *g* and divided into cytoplasmic (supernatant) or mitochondrial (pellet) fractions.

### Immunohistochemistry

To detect BMCC1 expression in normal human tissues, a tissue array containing paraffin-embedded tissue sections of organs from normal subjects and patients without cancer (AC1; SuperBioChips, Seoul, South Korea) was used. Skin (CX2), prostate (CA4) and colon (CDA3) tissue arrays (SuperBioChips) were used to detect BMCC1 in normal and tumor tissues. After the removal of paraffin, the slides were incubated with BMCC1 antibody (1 : 100 dilution) and subsequently incubated with a biotinylated universal secondary antibody (Vector Laboratories, Burlingame, CA, USA) (1 : 200 dilution). Immunocomplexes were visualized using an avidin–biotin immunoperoxidase system (Vector Laboratories). Hematoxylin (Sakura Finetek, Tokyo, Japan) was used as a counterstain.

### Statistical analysis

Quantitative experiments were independently performed at least three times. Error bars in each plot represent S.D. Statistical analysis was carried out and *P*-value was calculated using Excel software (Microsoft, Redmond, WA, USA).

## Abbreviations

ADR: adriamycin
ALK: anaplastic lymphoma kinase
ATM: Ataxia telangiectasia-mutated
BCH: BNIP2 and Cdc42GAP homology
BH3: BCL2-homology domain 3
BMCC1: BNIP2 and Cdc42GAP homology motif-containing molecule at the carboxyl-terminal region 1
CDDP: cisplatin
FOXO3a: forkhead box-O3a
NB: neuroblastoma
NGF: nerve growth factor
PARP1: poly (ADP-ribose) polymerase 1
PI3K: phosphoinositide 3-kinase
PIP5K: 1-phosphatidylinositol-4-phosphate 5-kinase
PtdIns: phosphatidylinositol
PTEN: phosphatase and tensin homolog
RhoGEF: RhoA-specific guanine nucleotide exchange factor
ROCK: Rho-associated, coiled-coil containing protein kinase
RT-PCR: reverse transcription polymerase chain reaction
