# Supplementary figures and images for: BMCC1, which is an interacting partner of BCL2, attenuates AKT activity, accompanied by apoptosis
Source: Cell Death Dis. 2015 Jan 22;6(1):e1607–. doi: 10.1038/cddis.2014.568 (PMC4669766; doi:10.1038/cddis.2014.568)

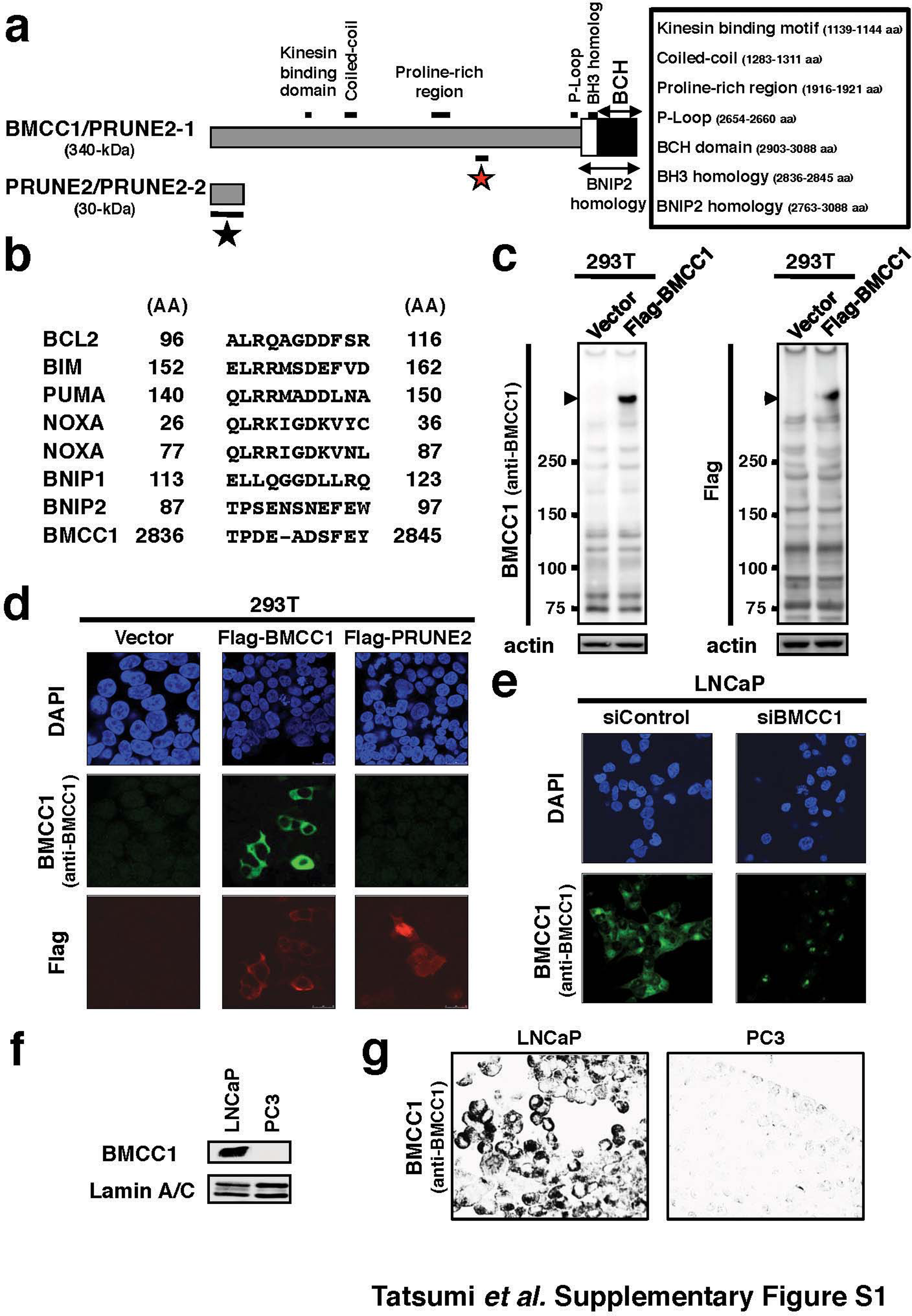

Supplement: Supplementary Figure S1 [file cddis2014568x1.tif]

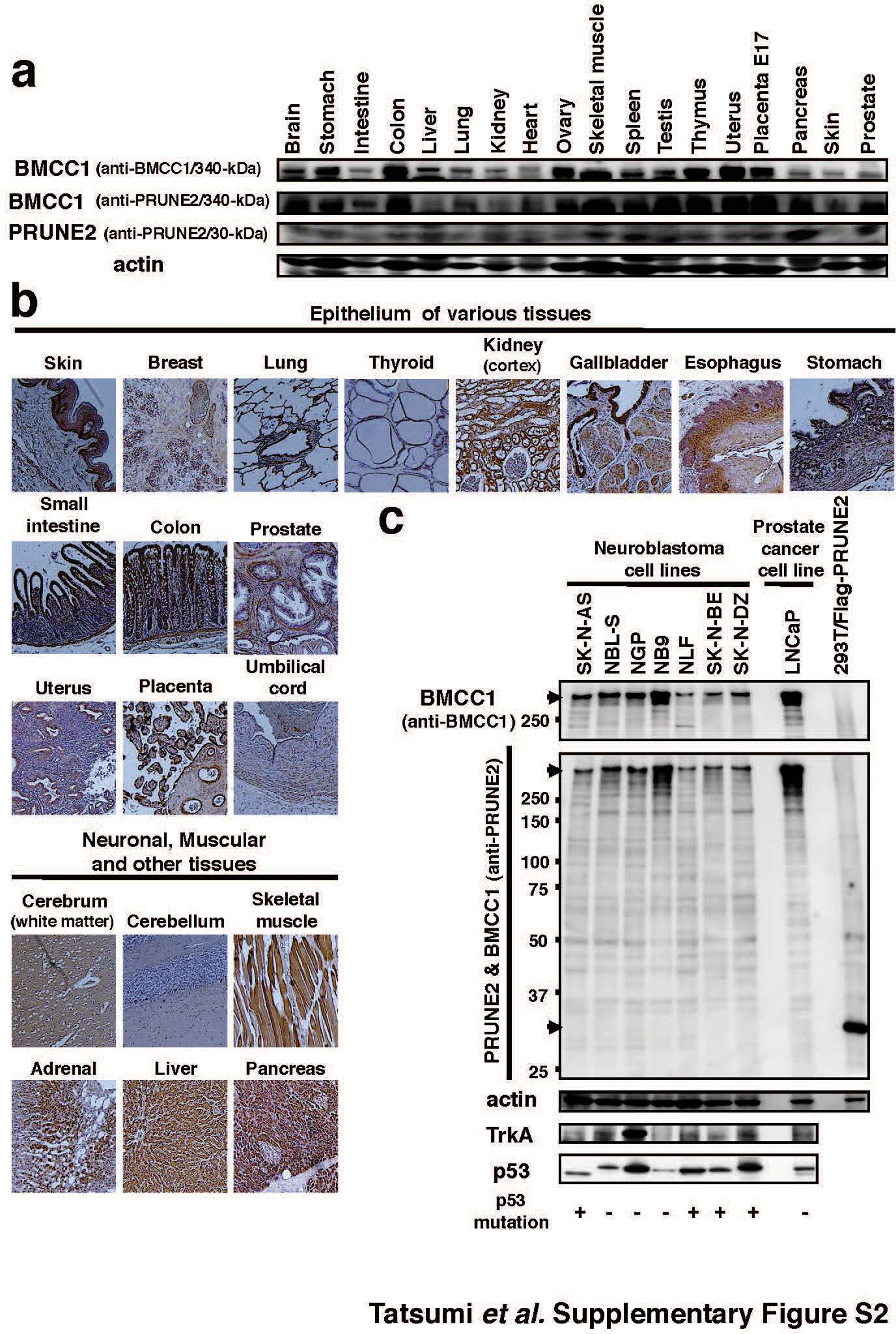

Supplement: Supplementary Figure S2 [file cddis2014568x2.tif]

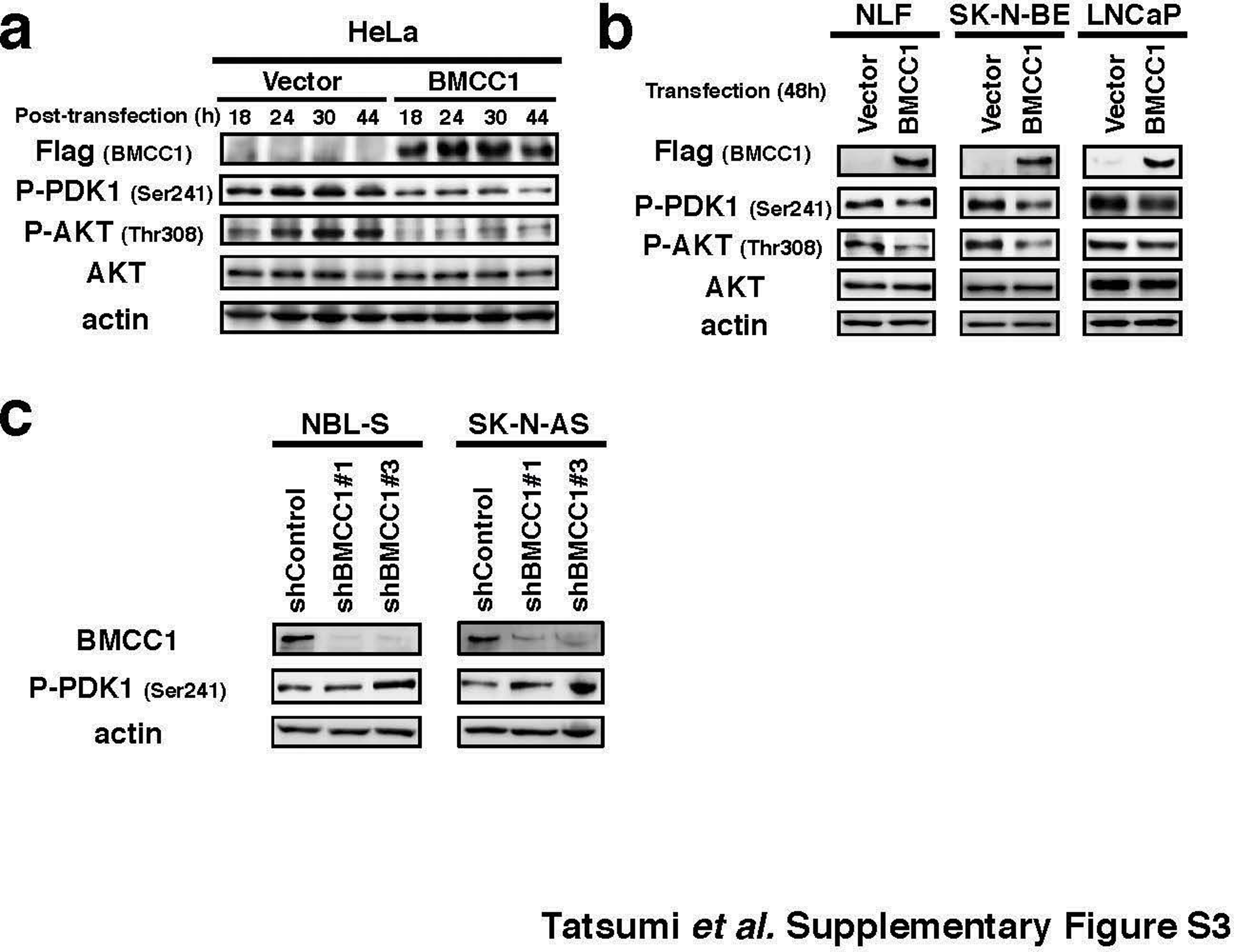

Supplement: Supplementary Figure S3 [file cddis2014568x3.tif]

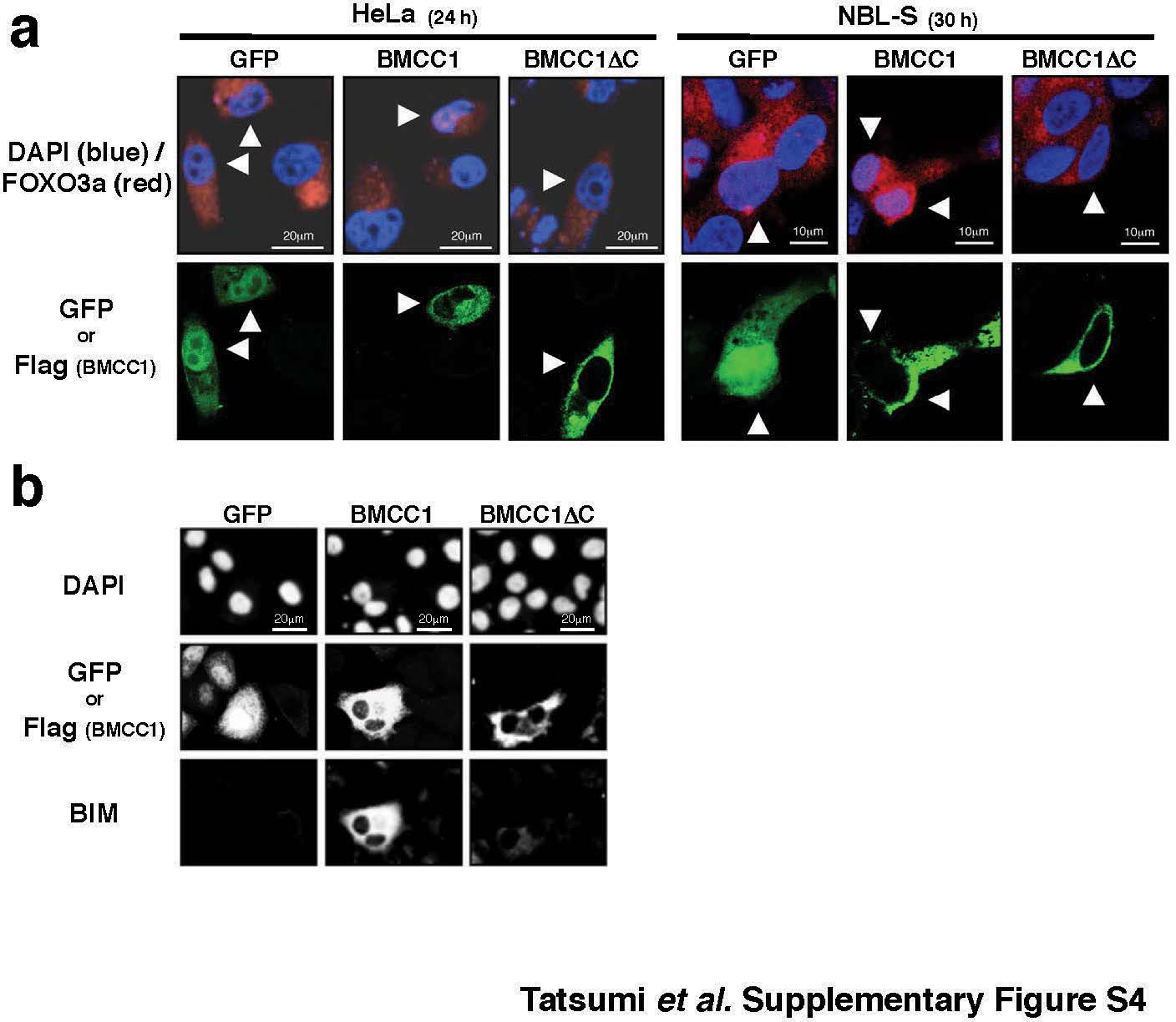

Supplement: Supplementary Figure S4 [file cddis2014568x4.tif]

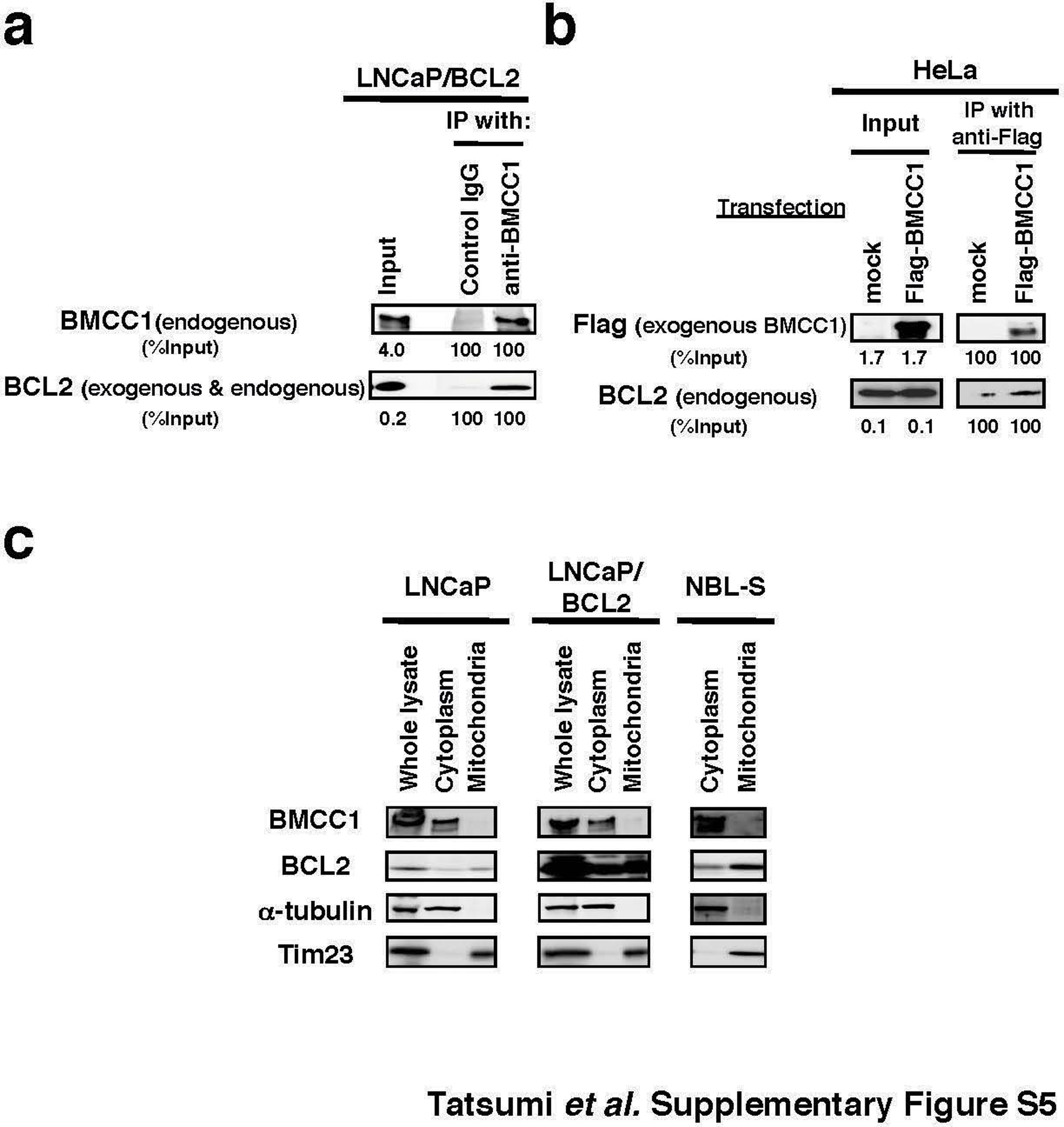

Supplement: Supplementary Figure S5 [file cddis2014568x5.tif]

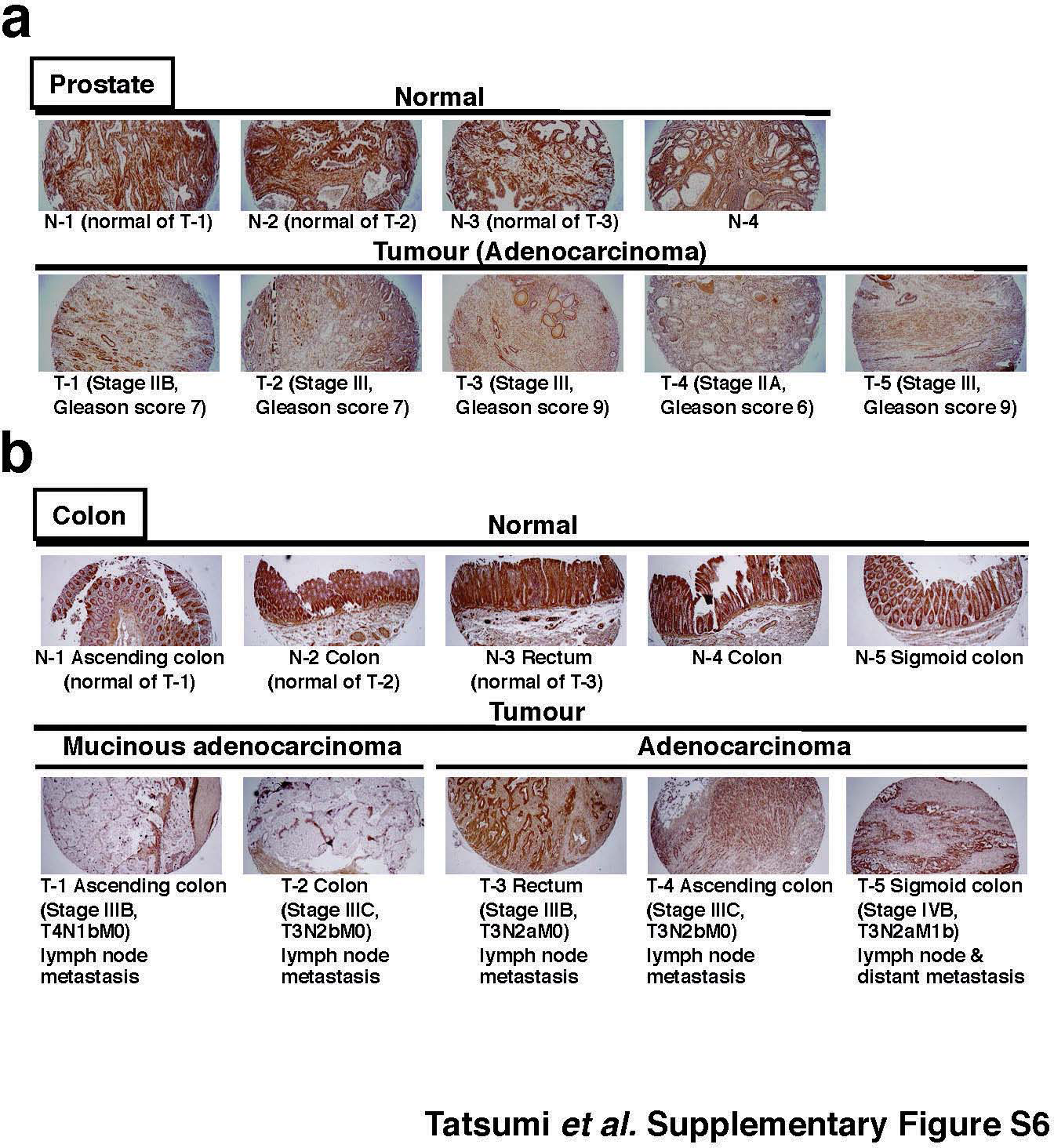

Supplement: Supplementary Figure S6 [file cddis2014568x6.tif]
